# Emerin is necessary for microtubule-organizing center translocation to the nuclear envelope of muscle cells

**DOI:** 10.1038/s41419-026-08819-6

**Published:** 2026-05-12

**Authors:** Elisabetta Mattioli, Vittoria Cenni, Patrizia Sabatelli, Elisa Schena, Spartaco Santi, Chiara Fiorillo, Claudio Bruno, Antonella Pini, Melania Giannotta, Marco Cavallo, Costantino Errani, Eleonora Cattin, Daniela Benati, Alessandra Recchia, Giovanna Lattanzi

**Affiliations:** 1 CNR Institute of Molecular Genetics “Luigi Luca Cavalli-Sforza”, Unit of Bologna, Bologna, Italy; 2 IRCCS Istituto Ortopedico Rizzoli, Bologna, Italy; 3 Department of Neurosciences, Rehabilitation, Ophthalmology, Genetics, Maternal and Child Health (DINOGMI), University of Genova, Genova, Italy; 4 Child Neuropsychiatry, IRCCS Istituto Giannina Gaslini, Genova, Italy; 5 Center of Translational and Experimental Myology, IRCCS Istituto Giannina Gaslini, Genova, Italy; 6 Child Neurology and Psychiatry Unit, IRCCS Istituto delle Scienze Neurologiche di Bologna, Bologna, Italy; 7 Shoulder-Elbow Surgery Unit, IRCCS Istituto Ortopedico Rizzoli, Bologna, Italy; 8 3rd Orthopedic and Traumatologic Clinic, Prevalently Oncologic, IRCCS Istituto Ortopedico Rizzoli, Bologna, Italy; 9 Department of Life Sciences, University of Modena and Reggio Emilia, Modena, Italy; 10 Center for Rigenerative Medicine, “Stefano Ferrari” University of Modena and Reggio Emilia, Modena, Italy

**Keywords:** Mechanisms of disease, Microtubules

## Abstract

During myogenic differentiation, the Microtubule-Organizing Center (MTOC) is relocated to the nuclear envelope by a molecular platform including Linker of Nucleoskeleton and Cytoskeleton (LINC) complex proteins, A Kinase Anchoring Proteins (AKAP9 and AKAP6) and Pericentriolar Material 1 (PCM-1). Here, we show that emerin is required for centrosomal protein recruitment to the nuclear periphery of myonuclei and microtubule dynamics. In fact, in type 1 Emery-Dreifuss Muscular Dystrophy (EDMD1), loss of emerin was associated with altered pericentrin recruitment to the nuclear envelope, LINC protein impairment at the nuclear poles of myonuclei and microtubule organization defects. As a consequence, dynein, mitochondrial distribution and nuclear alignment along the longitudinal axis of the myotubes were altered in EDMD1 myotubes. Moreover, reduced levels of AKAP6 and PKA were detected at the nuclear periphery of EDMD1 myotubes, possibly contributing to an aberrant nuclear localization of the mechanosensing factor YAP. Upon rescue of emerin expression by CRISPR correction of mutated *EMD* gene: SUN1/2, pericentrin, AKAP6 and PKA were restored at the nuclear envelope and a correct YAP localization was observed in EDMD1 muscle cells. These results show that emerin is required for Nuclear Envelope-MTOC (NE-MTOC) organization in differentiating skeletal muscle cells and suggest that disruption of such complex is a key pathogenetic event in Emery-Dreifuss Muscular Dystrophy.

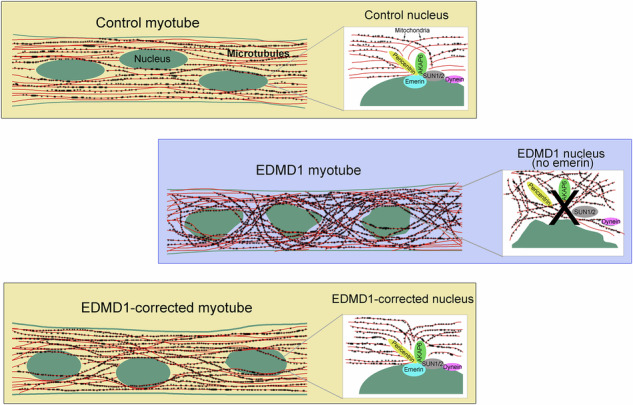

## Introduction

When myoblasts start the differentiation program, the centrosome is disrupted and there is a complete reorganization of MTOC at the nuclear envelope, where centrosomal proteins translocate to drive microtubule nucleation and nuclei repositioning [1–3]. In muscle cells, microtubules are in fact redistributed in arrays parallel to the longitudinal axis of the cell and their organization is essential to distribute nuclei along the length of the myotubes [4]. Microtubule nucleation at the nuclear envelope, requires a synchrony between proper MTOC repositioning and Golgi apparatus reorganization, and involves different A-kinase anchoring proteins (AKAPs), a group of proteins able to recruit cAMP-dependent protein kinase (PKA) close to its substrates [5]. Muscle-specific AKAP (AKAP6 or mAKAP) is a key component of the myotube NE-MTOC. AKAP6 is exclusively expressed in striated muscle cells and it is required for skeletal muscle differentiation [6, 7]. AKAP6 connects the Golgi apparatus to the nucleus through AKAP9, another A-kinase anchoring protein able to regulate microtubule dynamics and nuclear positioning in rat cardiomyocytes and muscle cells [2, 4]. It has been demonstrated that AKAP6 acts as an adaptor tying both AKAP9 and nesprin1 and is necessary for the AKAP9 nuclear recruitment [2]. These interactions are required to anchor the Golgi apparatus to the nuclear envelope [2]. In differentiating myoblasts, AKAP6 not only binds PKA and the ryanodine receptor at the nuclear membrane, but also it interacts with several enzymes such as PP1, PP2 and ERK5 contributing to signaling pathways [8, 9]. Moreover, at the nuclear envelope level, AKAP6 forms complexes including PKA and histone deacetylases (HDACs), thus regulating HDACs phosphorylation and activity [10, 11].

A main driver of nuclear envelope-related mechanisms in developing skeletal muscle and centrosome positioning, is the LINC complex, a protein platform encompassing the perinuclear space, and the inner nuclear membrane and establishing dynamic contacts with the nuclear lamina on one side and the cytoskeleton on the other side [12, 13]. Nesprin1α, a LINC complex component, is involved in centrosomal protein recruitment to the nuclear surface and repositioning of nuclei during myotube formation, via AKAP9 [4, 7, 14–16]. Moreover, nesprin1α anchors AKAP6 on the nuclear rim in cardiac myocytes [17]. Interestingly, in patient myotubes carrying mutations in *SYNE* 1 gene, encoding nesprin1, microtubule nucleation and nuclear positioning defects have been demonstrated [4]. Particularly important for the connection between cytoskeleton and nucleoskeleton is the axis nesprin1α, emerin, lamin A/C, which is disrupted in human dermal fibroblasts of Emery-Dreifuss Muscular Dystrophy patients with *SYNE* mutations [18]. Emerin, an integral protein of the inner nuclear membrane, is another important member of the LINC complex and it is linked to EDMD1, a muscular dystrophy associated with early joint contractures, muscular weakness and cardiomyopathy [19, 20]. Emerin establishes relationships with other LINC complex proteins [18, 21] and it is phosphorylated by protein kinase A on serine residues during muscle differentiation [22, 23]. It has been demonstrated that emerin influences centrosome localization and orientation [24, 25]. An increase of the distance between the nucleus and the centrosome was, in fact, observed in emerin-null human dermal fibroblasts and myoblasts [24, 26]. Emerin was also detected in the ER and Golgi compartments, directly bound to β-tubulin [24]. Furthermore, it has been published that emerin knockdown induces cytoskeletal perturbations, with defects in Golgi apparatus and MTOC localization [26]. An extra nuclear emerin localization, associated with mitotic spindle microtubules and centrosomes, was also observed in HeLa cells [27]. Moreover, cytoplasmic emerin was detected in human myotubes, in association with actin [28, 29]. Interestingly, also lamin A/C and SUN1, whose mutations cause EDMD2 and an EDMD-like phenotype, respectively, are involved in MTOC translocation and pericentrin relocalization, and NE-MTOC defects were observed in laminopathic myotubes from *LMNA* or *SUN1*-linked muscular dystrophy [30, 31].

In this study, we observed that emerin interacts with pericentrin, AKAP6 and PKA, during muscle differentiation. Interestingly, we observed a perturbation of NE-MTOC in EDMD1 differentiating myoblasts that lack emerin. In those cells, pericentrin, AKAP6 and PKA were not properly recruited to the nuclear envelope and microtubule organization was altered. We suggest that emerin could regulate centrosomal proteins through SUN1 and SUN2. These LINC complex proteins were delocalized in EDMD1-myotubes and rescued along with centrosomal proteins after re-expression of emerin by CRISPR editing. Our results, shed light on a new functional role of emerin in MTOC relocalization to the nuclear envelope during muscle differentiation and suggest that defects in this mechanism could be involved in the onset of EDMD1 phenotype.

## Results

### Emerin interacts with pericentrin and AKAP6 during skeletal muscle differentiation

**Fig. 1:**
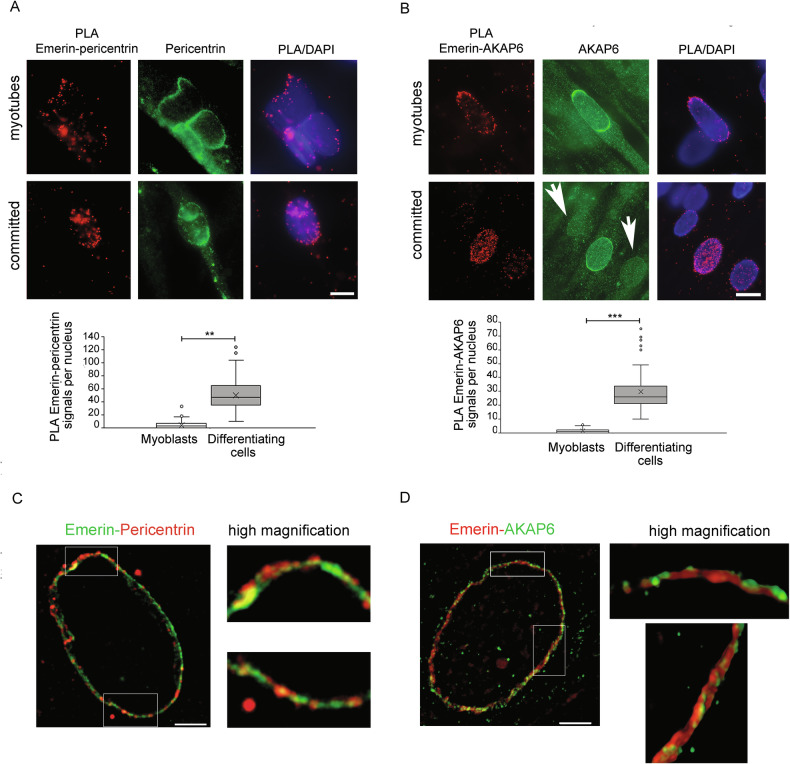
Emerin binds AKAP6 and pericentrin in differentiated muscle cells. **A** Committed myoblasts (committed) and myotubes (myotubes) from healthy donors were subjected to proximity ligation assay (PLA) of emerin and pericentrin (red dots), and immunofluorescence analysis (IF) of pericentrin (green). Right panel: merged image of PLA between emerin and pericentrin and DAPI (PLA/DAPI). Quantitative analysis of PLA signals was reported in the lower graph. **B** PLA between emerin and AKAP6, IF of AKAP6 (green) and merged image of PLA emerin and AKAP6 and DAPI (PLA/DAPI). White arrows indicate not differentiating myoblasts. Statistical analysis of PLA signals was reported in the lower graph. **C** Structured illumination microscopy (SIM) of emerin (green) and pericentrin (red), high magnification of the white boxes was reported on the right. **D** SIM analysis of emerin (red) with AKAP6 (green), high magnification of the areas in the white boxes is shown on the right. Scale bars, 10 μm for figures (**A**, **B**) and 2 μm for figures (**C**, **D**). DAPI (blue staining) was used to counterstain cell nuclei in the figures (**A**, **B**). Data are represented as a box blot graph of three independent experiments (*n* = 3) and *n* = 25 cells for each sample; statistically significant differences between values are indicated with Student’s *t* test (**p < 0.01, ***p < 0.001).

Given the previously reported involvement of emerin in the regulation of centrosome positioning [24], we decided to investigate emerin interaction with MTOC proteins in differentiating human muscle cells from healthy donors. As reported [6, 31] we observed pericentrin and AKAP6 recruitment to the nuclear periphery during myoblast differentiation (Figure. S1). Here, proximity ligation assay (PLA) demonstrated that emerin interacts with pericentrin already in committed myoblasts, and this binding persists in myotubes (Fig.1A). Moreover, we observed emerin-AKAP6 complexes in differentiating human muscle cells (Fig. 1B). Specificity of emerin-AKAP6 binding was validated by the absence of PLA signals in cycling myoblasts, which do not express AKAP6 (Fig. 1B, white arrows). To confirm the relationship between pericentrin, AKAP6 and emerin, we performed structured illumination microscopy (SIM) analysis in human myotubes, which showed emerin-centrosomal protein “contact areas” at the nuclear periphery (Fig.1C, D).

These results demonstrated emerin binding to pericentrin and AKAP6 and allowed us to hypothesize that emerin might be involved in NE-MTOC recruitment to the nuclear surface during muscle differentiation.

### Loss of emerin determines pericentrin and AKAP6 defective recruitment to the nuclear envelope of EDMD1-myotubes and EDMD1 muscle tissue

**Fig. 2:**
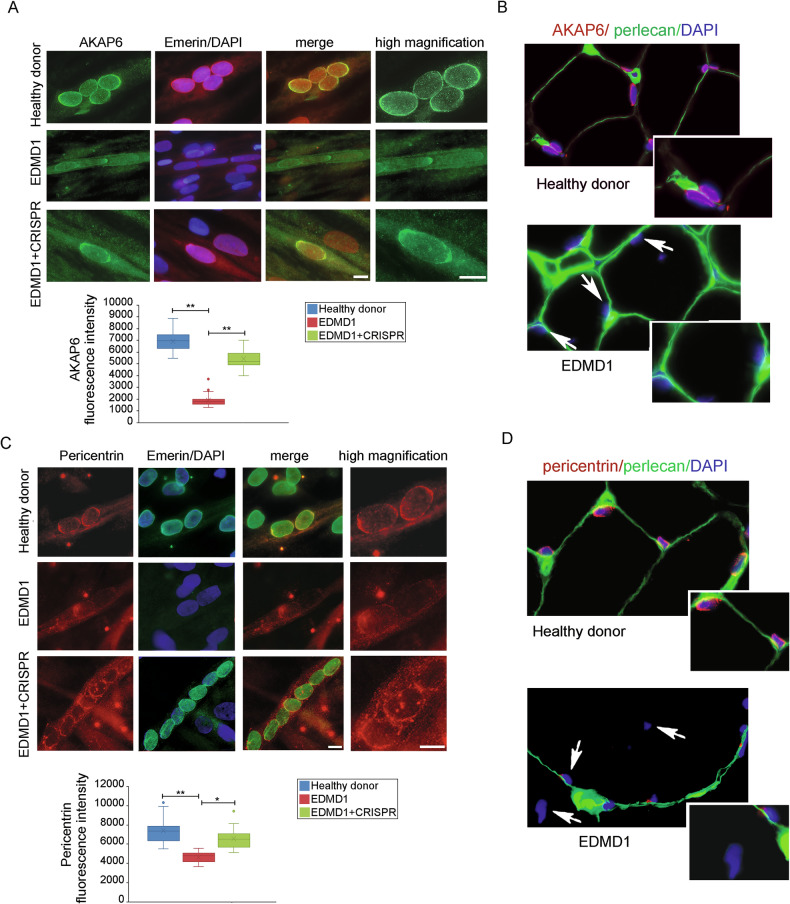
Reduced AKAP6 and pericentrin recruitment to the nuclear rim in EDMD1-myotubes and skeletal muscle sections. **A** Immunofluorescence analysis of AKAP6 (green) and emerin (red) in myotubes from healthy donors, EDMD1 patients and CRISPR-edited EDMD1 (EDMD1 + CRISPR). The right panels show high magnification. Quantitative analysis of mean fluorescence intensity of AKAP6 signal is reported in the graph. **B** Immunofluorescence analysis of AKAP6 (red) and perlecan (green) in skeletal muscle sections of healthy control and EDMD1 muscle. White arrows indicate nuclei lacking AKAP6 signal. **C** IF of pericentrin (red) and emerin (green) in healthy donors, EDMD1-myotubes and EDMD1 + CRISPR. High magnification is shown in the right panels. Quantitative analysis of pericentrin mean fluorescence intensity is reported in the graph below. **D** Immunostaining of pericentrin (red) and perlecan (green) in skeletal muscle sections of healthy control and EDMD1 muscle. White arrows indicate nuclei that lost pericentrin signal. DAPI (blue) was used to counterstain cell nuclei. Scale bars, 10 μm. Data are represented as a box blot graph of three independent experiments (*n* = 3), and *n* = 20 cells for each sample were counted. Statistically significant differences between values calculated by Student’s *t* test are indicated by asterisks (**p < 0.01 or *** *p* < 0.001 or ****p < 0.0001).

Considering our previous results, demonstrating NE-MTOC defects in myotubes from *SUN1*-linked muscular dystrophy, where emerin-SUN1 interaction was disrupted [31] we decided to investigate pericentrin and AKAP6 in emerin-null EDMD1-myotubes. Immunofluorescence analysis of AKAP6 revealed impaired recruitment to the nuclear periphery in EDMD1myotube nuclei (Fig. 2A). Interestingly, when emerin expression was restored in EDMD1 myotubes through *EMD* editing by CRISPR, we observed a complete recovery of AKAP6 recruitment at the nuclear periphery (Fig. 2A lower panels). Immunolabeling with secondary-only antibody excluded any non-specific signal (Figure. S2A). This result was also confirmed by western blotting analysis (Figure. S2B and Supplementary file). Importantly, we also investigate AKAP6 in skeletal muscle tissue and we identified strongly reduced levels of AKAP6 in EDMD1 mature muscle fibers (Fig. 2B and Figure S2C).

Furthermore, we observed reduced levels of pericentrin at the nuclear envelope of EDMD1 myotubes (Fig. 2C). However, when emerin expression was restored by CRISPR system, pericentrin nuclear localization was recovered in myotubes (Fig. 2C lower panels and S2D). Notably, reduced pericentrin staining at the nuclear rim was also found in EDMD1 skeletal muscle fibers (Fig. 2D and Figure S2E). The roles of AKAP6 and pericentrin in skeletal muscle tissue warrant further investigation.

Efficiency of *EMD* gene correction in caveolin 3-positive EDMD1 myotubes was about 30% as shown in Figure. S3A. Differentiation index and fusion index were reduced in EDMD1 myotubes and improved after *EMD* gene correction (Figure. S3B). The CRISPR-mediated reframing strategy used in EDMD1 #2 myoblasts resulted in a shorter emerin protein lacking seven amino acids (L, G, Q, D, R, Q, V from aa 217 to 223) just upstream of the C- terminal transmembrane hydrophobic domain. To the best of our knowledge, those missing amino acids have not been associated with a functional domain [32]. Here, we show that emerin protein lacking this region still preserves the functionality to recruit pericentrin and AKAP6 to the nuclear rim in differentiated myotubes.

These experiments supported the finding that emerin is required for NE-MTOC formation in human myotubes.

### Defective PKA recruitment to the nuclear envelope and YAP nuclear accumulation in EDMD1-myotubes

**Fig. 3:**
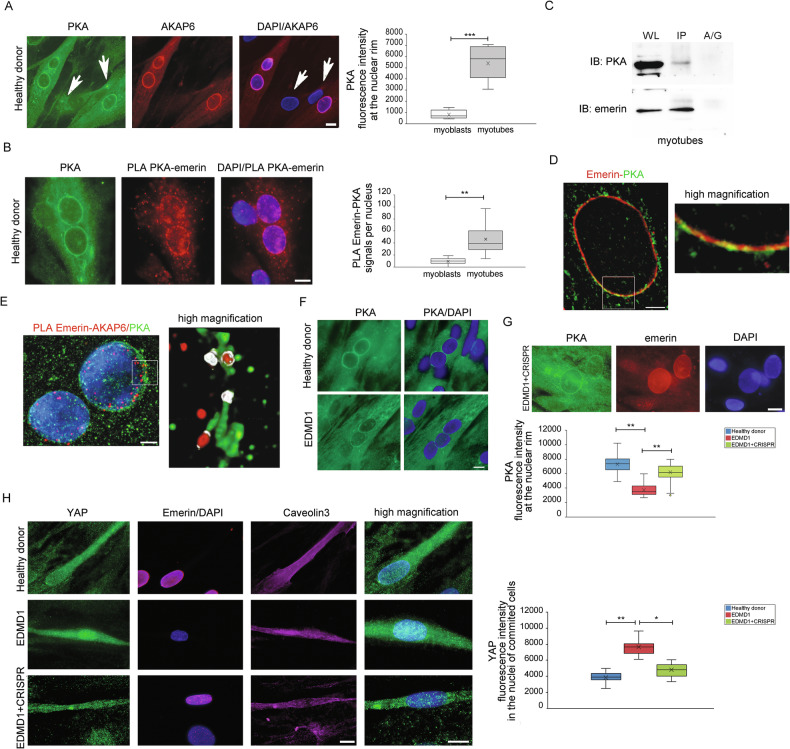
Emerin interacts with PKA at the nuclear envelope of human myotubes. **A** Double IF of PKA RII subunit (green) and AKAP6 (red) in myotubes from healthy donor. In the first picture, white arrows identified centrosome in non-differentiated myoblasts. On the right, AKAP6 and DAPI staining (blue) identified non-differentiated myoblasts (white arrows). Mean of fluorescence intensity of PKA at the nuclear rim is reported in the graph. **B** IF of PKA RII subunit (green) and PLA of PKA and emerin in control myotubes (red dots). PLA of PKA and emerin, and DAPI (blue staining) are merged in the right picture. Quantitative analysis of PLA signals is reported in the graph. **C** Whole lysate of healthy donor muscle cells after 6 days of differentiation, was immunoprecipitated using antibody against emerin (IP) and immunoblotted (IB) for PKA and emerin. A/G is negative control without anti-emerin antibody; WL, whole lysate. **D** SIM analysis of emerin and PKA in a representative nucleus of a control myotube. High magnification of a white box was reported on the right. **E** SIM analysis of PLA between emerin and AKAP6 (red dots) and IF of PKA (green). High magnification of a white box is reported on the right; white signals are the contact zones. **F** Immunofluorescence analysis of PKA (green) in control and EDMD1 myotubes. **G** Immunofluorescence analysis of PKA (green) and emerin (red) in gene-corrected EDMD1 myotubes. Mean fluorescence intensity of PKA at the nuclear rim is reported in the graph below. **H** Immunofluorescence analysis of YAP (green), emerin (red) and caveolin 3 (violet) in healthy control and EDMD1 committed myoblasts edited (EDMD1 + CRISPR) or not with CRISPR system. High magnification is reported on the right panel. Mean fluorescence intensity of YAP nuclear localization is reported in the graph on the right. DAPI (blue) was used to counterstain cell nuclei in the figures (**A**, **B**, **E**–**H**). Scale bars 10 μm for all the figures and 2 μm for SIM analysis. Data are represented as a box plot (*n* = 30 cells for condition) of three independent experiments (*n* = 3), and statistically significant differences between values calculated by Student’s *t* test are indicated by asterisks (*p < 0.05*,* **p < 0.01, ***p < 0.001).

AKAPs function is to anchor PKA to specific cellular compartments to regulate the phosphorylation of its substrates [33]. Our results demonstrate that in control cycling myoblasts, PKA is localized in the centrosome and in the endoplasmic reticulum (Fig. 3A, first picture, white arrows). However, during myoblast differentiation, PKA is recruited to the nuclear envelope, along with AKAP6 (Fig. 3A and Figure S4). To understand the relation between emerin and PKA relocalization to the NE, we decided to investigate emerin-PKA interaction in control myotubes. PLA demonstrated that emerin directly interacts with PKA RII subunit at the nuclear envelope of myotubes (Fig. 3B). PKA and emerin was also co-immunoprecipitated in lysates of differentiated muscle cells (Fig. 3C and Supplementary file). Immunoprecipitation with an antibody against emerin, showed that only a small fraction (18%) of the total PKA bound emerin (Fig. 3C). An emerin-PKA-containing complex was lastly confirmed by SIM analysis (Fig. 3D). Furthermore, a ternary complex including, PKA, emerin and AKAP6 was demonstrated by SIM in nuclei of healthy donor myotubes (Fig. 3E). White signals are generated by colocalization of PLA signal (interaction between emerin and AKAP6) and PKA staining (Fig. 3E). Interestingly, the reduction of PKA recruitment to the nuclear periphery was reduced in EDMD1 myotubes and restored after CRISPR-mediated *EMD* editing (Figs. 3F, G). Since it has been demonstrated that PKA is able to inhibit nuclear accumulation of Yes-associated protein (YAP) in muscle cells, thus preventing expression of Hippo-target genes [34], we decided to investigate YAP localization in control and EDMD1 muscle cells. YAP was retained in the nucleus of committed EDMD1 myoblasts, showing defective PKA nuclear localization, whereas YAP cytoplasmic localization was restored in *EMD* gene edited myoblasts expressing emerin (Fig. 3H). These findings demonstrated that emerin is necessary for a correct PKA localization to the nuclear envelope of myotubes, probably through AKAP6 binding and this protein platform influences YAP localization.

### SUN1 and SUN2 mislocalization in myotubes lacking emerin

**Fig. 4:**
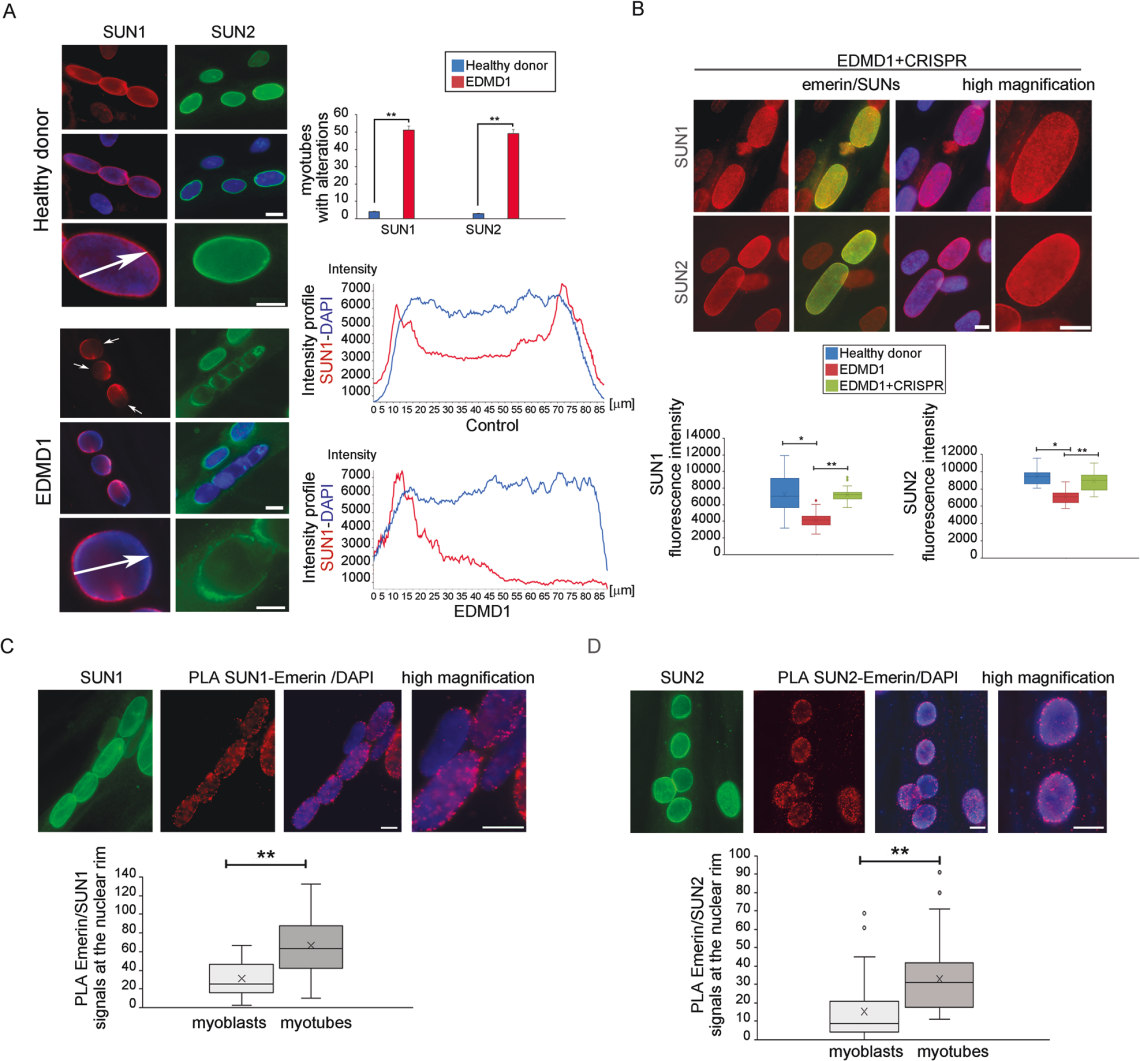
SUN1 and SUN2 mislocalization in EDMD1-myotubes nuclei. **A** Immunofluorescence analysis of SUN1 (red) and SUN2 (green) in myotubes from a healthy donor (Healthy donor) and EDMD1 myotubes (EDMD1). High magnification was shown in the lower panels. Percentage of myotubes with alteration of LINC complex proteins is reported in the graphs on the right (*n* = 50 myotubes for sample were counted). Fluorescence intensity profile of SUN1 (red) in a representative control and EDMD1 myotube nuclei (white arrow) is reported in the graphs on the right. **B** IF of SUN1 (red) and SUN2 (red) and merge image with emerin in gene-corrected EDMD1 myotubes. Statistical analysis of fluorescence intensity of SUN1 and SUN2 is reported in the lower graphs. **C** Immunolabeling of SUN1 (green) and PLA analysis of SUN1 and emerin (red dots) in healthy donor myotubes; high magnification of PLA signals and DAPI (high magnification) are shown in the right panel. Quantitative analysis of PLA signals on the rim is reported in the lower graph. **D** IF of SUN2 (green) and PLA analysis of SUN2 and emerin (red dots) in control myotubes. High magnification of PLA signal (red) and DAPI (blue) were reported on the right panel. Statistical analysis of the PLA signals on the nuclear envelope was reported in the lower graph. DAPI (blue) was used to counterstain nuclei. Scale bars, 10 μm. Data are means ± SD of three independent experiments (*n* = 3), are represented as box plot graphs (*n* = 25 myotubes per sample). Statistically significant differences between values calculated by Student’s *t* test are indicated by asterisks (*p < 0.05, **p < 0.01,*** *p* < 0.001).

It has been demonstrated that LINC complex proteins, such as SUN1 and SUN2, are important to drive relocalization of MTOC to the nuclear periphery [1, 4]. Since emerin interacts with both SUN1 and SUN2 [31], we decided to investigate SUN proteins. By immunofluorescence analysis, we observed that SUN1 and SUN2 were mislocalized in 50% of EDMD1-myotubes nuclei (Fig. 4A). SUN1 was absent from the nuclear poles in emerin-null myotubes (Fig. 4A, white arrows), while emerin expression by CRISPR editing restored SUN1 and SUN2 localization (Fig.4B). These findings demonstrated that emerin is important for proper LINC complex protein localization at the nuclear poles of muscle cells. In support of this, we observed emerin interaction with SUN1 and SUN2 both in myoblasts and myotubes (Fig. 4C, D). Interestingly, in healthy donor myotube nuclei, PLA signals were enriched at the nuclear periphery as compared to undifferentiated myoblasts (Fig. 4C, D). These results demonstrated the existence of a tight network of interactions between emerin and SUN proteins in differentiating myoblasts, possibly involved in MTOC formation at the nuclear envelope poles.

### Emerin deficiency affects the microtubule system and nuclear dynamics in EDMD1myotubes

**Fig. 5:**
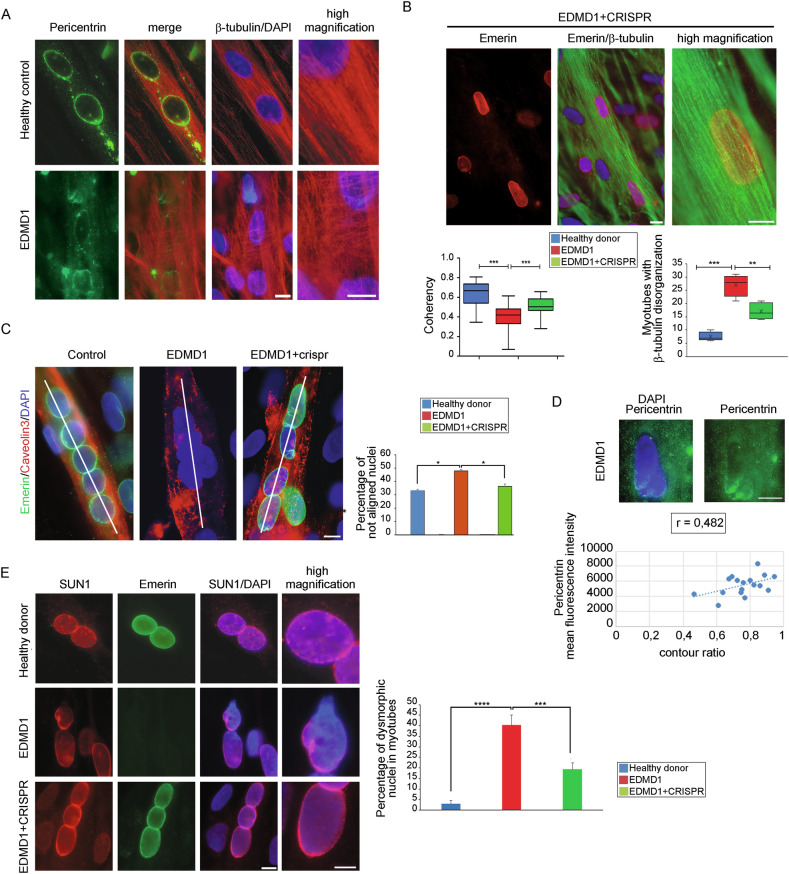
Microtubules disorganization, misaligned and dysmorphic nuclei were observed in EDMD1 myotubes. **A** Immunolabeling of pericentrin (green) and β-tubulin (red) in healthy donor (Healthy donor) and EDMD1-myotubes (EDMD1). High magnification was reported in the right panels. **B** IF of emerin (red) and β-tubulin (green) in EDMD1 myotubes subjected to CRISPR editing (EDMD1 + CRISPR). High magnification was reported in the right panel. Coherency of β-tubulin and percentage of myotubes with β-tubulin disorganization, were reported in the lower graphs (*n* = 30 myotubes for sample were counted). **C** Immunostaining of emerin (green) and caveolin 3 (red) in control, EDMD1 and EDMD1 + CRISPR myotubes. In the graph on the right, the percentage of nuclei not aligned along the longitudinal axis of the myotube. **D** IF of pericentrin (green) and DAPI (blue) in EDMD1 nucleus. Statistical analysis of correlation between pericentrin fluorescence intensity and contour ratio is shown in the right graph. *r*, indicated Pearson correlation coefficient. **E** Immunostaining of SUN1 (red) and emerin (green) in myotubes from: healthy donor, EDMD1 and EDMD1 after CRISPR editing. High magnification was reported in the panels on the right, and quantitative analysis was reported on the right graph (*n* = 50 nuclei for sample were counted). DAPI (blue) was used to counterstain cell nuclei. Scale bars, 10 μm. Box plot are represented of plotting individual data points of three independent experiments (*n* = 3), and statistically significant differences between values are indicated with Student’s *t* test (*p < 0.05 or **p < 0.01,*** *p* < 0.001, **** *p* < 0.0001) or Pearson test.

It is known that centrosomal protein recruitment to the nuclear envelope is essential for a correct microtubule network formation. Since centrosomal proteins were not properly localized in EDMD1 myotube nuclei, we sought to investigate microtubule organization in EDMD1myotubes. In control myotubes, microtubules ran parallel to the major myotube axis, as expected [4]. However, in EDMD1-myotubes showing low pericentrin levels at the nuclear envelope, we observed a random intertwining of microtubules (Fig. 5A), while microtubule organization was rescued in EMD gene-corrected EDMD1 myotubes (Fig. 5B). Quantification of microtubule orientation is reported as a measure of coherency in Fig. 5B, where coherency indicates the degree to which the microtubules are oriented. Since nuclear movement and alignment during myotube formation are dependent on MTOC recruitment to the nuclear envelope and microtubule structure [15], we measured the alignment of nuclei along the longitudinal axis of the myotubes. We observed that 48% of the nuclei of EDMD1 myotubes were not aligned, while in control myotubes, most of the nuclei were correctly distributed. Following emerin recovery by CRISPR correction, the percentage of mislocalized nuclei in EDMD1 myotubes was decreased to 35% (Fig. 5C). In EDMD1 myotubes, we also observed a high percentage of dysmorphic nuclei which correlates with pericentrin reduction at the nuclear envelope of EDMD1-myotubes (Fig. 5D). Pearson correlation coefficient (r) indicated a moderate positive correlation between contour ratio and pericentrin fluorescence intensity (Fig. 5D). Finally, nuclear morphology was rescued in EDMD1 myotubes subjected to CRISPR *EMD* editing (Fig. 5E). These results demonstrated that defective MTOC protein recruitment to the nuclear envelope due to emerin absence in EDMD1 myotubes, affects microtubule organization, nuclear alignment and nuclear morphology.

### Defective dynein localization and mitochondrial distribution in EDMD1 myotubes

**Fig. 6:**
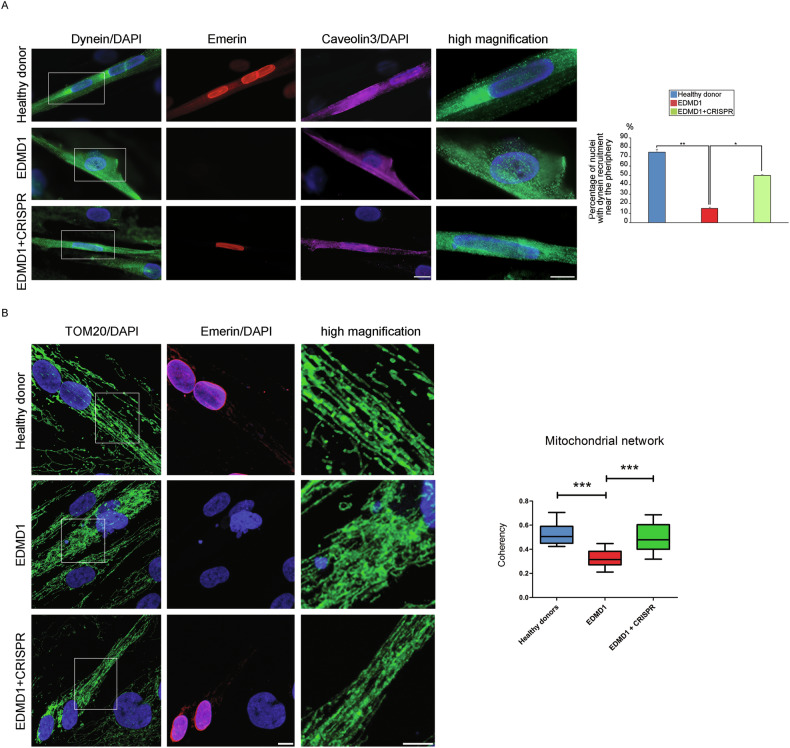
Dynein localization and mitochondrial network were altered in EDMD1 myotubes. **A** Immufluorescence of dynein (green), emerin (red) and caveolin3 (violet) in myotubes of healthy control (healthy donor), EDMD1 (EDMD1) and EDMD1 + CRISPR. High magnification of the white box was shown in the right panel, and statistical analysis was reported on the right. **B** Immunolabeling of TOM20 (green) and emerin (red) in myotubes of control, EDMD1 and EDMD1 subjected to CRISPR technology. High magnification (white boxes) was reported in the right panel. Coherency of mitochondrial network was reported in the graph on the right (*n* = 30 myotubes per sample were counted). DAPI (blue) was used to counterstain cell nuclei. Scale bars, 10 μm. Statistical analysis of three independent experiments (*n* = 3) and statistically significant differences between values are indicated by Student’s *t* test (*p < 0.05 or **p < 0.01,*** *p* < 0.001, **** *p* < 0.0001).

Nuclear positioning in myotubes further involves the microtubule motor proteins dynein and kinesin, which are recruited to the nuclear envelope by nesprin1 and PCM1, allowing nuclear rotation [15, 35]. Here, we observed that in healthy donor myotubes, dynein is distributed to a poles of the nucleus and aligns along the microtubules (Fig. 6A). However, in EDMD1 myotubes dynein is not enriched at the pole of to the nucleus and cytoplasmic localization is defective (Fig. 6A). A rescue of dynein distribution at the poles of the nuclei was observed in CRISPR-edited EDMD1 (Fig. 6A). Since dynein is a primary motor protein for mitochondria movement along microtubules [36], we decided to analyze by confocal microscopy, mitochondrial distribution in EDMD1 myotubes. We observed that, in healthy controls, mitochondria run on a microtubule system, along the longitudinal axis of the myotubes; whereas in EDMD1, where microtubules and dynein are defective, the mitochondrial network is lost and is restored in EDMD1 myotubes subjected to CRISPR correction (Fig. 6B). These findings demonstrated that emerin is related to dynein localization and mitochondrial distribution in myotubes.

## Discussion

In differentiating skeletal muscle cells, MTOC function is re-assigned to the nuclear envelope, and it is essential to drive microtubule nucleation and nuclear positioning during myotube formation [1]. It has been demonstrated that nesprin1α is the main driver of MTOC translocation to the nuclear envelope and other LINC components are also involved [4]. We previously reported that SUN1-mutated myotubes from an EDMD-like patient, show a reduced SUN1-emerin interaction and defective pericentrin recruitment to the nuclear rim, linked to altered nuclear positioning and microtubule nucleation defects [31]. Further, mislocalization of the nuclear envelope protein SAMP1, also involved in microtubule organization, has been observed at the nuclear poles of EDMD2 myotubes [37]. Finally, altered microtubule nucleation from the nuclear periphery of EDMD4 patient myotubes (carrying *SYNE* 1 mutation) has been demonstrated [4]. All these data strongly support a major role of defective nuclear MTOC-dependent microtubule dynamics in the pathogenesis of diverse EDMD forms. In this study, we demonstrated that such pathomechanism is also related to EDMD1. Here, we show that emerin interacts with pericentrin, AKAP6 and PKA and a functional emerin is necessary for proper MTOC protein recruitment to the nuclear envelope during muscle differentiation. In fact, in emerin-null myotubes, pericentrin and AKAP6 failed to properly translocate to the nucleus and LINC components SUN1 and SUN2 were mislocalized. Restoration of emerin expression through CRISPR correction, rescued NE-MTOC components recruitment to the nuclear envelope. Our results demonstrate that emerin is important to maintain the integrity of the LINC platform and direct centrosomal protein recruitment to the nuclear envelope, a process of fundamental importance for the nucleation of microtubules in myotubes [14]. In control myotubes, microtubules run parallel to the longitudinal axis of the cell, while defective NE-MTOC protein translocation was associated with microtubule disorganization in EDMD1 myotubes. These observations are consistent with data previously, published by Salpingidou et al. showing that emerin is involved in centrosome positioning through β-tubulin binding near the Golgi compartment [24]. Interestingly, we also observed alteration of both pericentrin and AKAP6 in EDMD1 muscle tissue, indicating a role of emerin in MTOC organization in mature muscle fibers. The role of such nuclear microtubule cage has been recently highlighted in an elegant study. The authors show that the LINC complex and lamin A/C mediate mechanical compressive forces exerted on the nucleus during myofiber maturation, while the microtubule cage preserves nuclear shape by counteracting, as an elastic force driver, myonuclear compression and elongation [38]. Here, we demonstrate that also emerin plays a relevant role in microtubule cage organization, as emerin absence in EDMD1 causes an altered microtubule network around nuclei. Of note, we found a good correlation between impaired pericentrin recruitment to the nuclear rim and nuclear dysmorphism. This result is in line with the possibility that an altered microtubule nucleation in EDMD1 myotubes might impinge on nuclear shape and, in turn, chromatin arrangement. On the other hand, our previous data showing that prelamin A and the LINC complex play a fundamental role in nuclear movement through modulation of microtubule dynamics [31], suggest that even emerin-driven mechanisms affecting the perinuclear microtubule cage, might impinge on localization of myotube nuclei. In support of this hypothesis, here we show a defective nuclear alignment in EDMD1 myotubes. Microtubule-directed nuclear movement is not only required during myotube formation, but also in muscle fiber repair upon physiological damage occurring during contraction [39]. Given the need of an integral MTOC for microtubule dynamics, pericentrin absence in mature muscle fibers, here reported for the first time in EDMD1, likely affects microtubule-dependent nuclear movement in a muscle repair context. As a whole, our data pose emerin, AKAP6 and pericentrin as fundamental organizers of the microtubule network around the nucleus and envisage a pathogenetic role of pericentrin loss and microtubule nuclear cage disruption in EDMD1. At the same time, the dynein mislocalization observed in EDMD1 myotubes may also contribute to myonuclear misalignment. Indeed, dyneins and kinesins are the primary regulators of nuclear positioning during myotube formation [15, 35]. Furthermore, we show that microtubule disorganization and dynein defects in EDMD1 myotubes, were also associated with alterations of mitochondrial network. Mitochondrial dysfunction has been reported in emerin-deficient cardiomyocytes and the relevance and timing of emerin-related events in mitochondrial network formation warrant further investigation [40].

It has been proved, that during muscle differentiation emerin is phosphorylated by several kinases including PKA [22, 23, 32]. In this study, we observed PKA nuclear localization and colocalization with AKAP6 in myotubes and we hypothesized that the AKAP6 could mediate PKA recruitment close to emerin in myotubes, leading to emerin phosphorylation. In fact, our results demonstrated the existence of a protein platform including emerin, PKA and AKAP6 at the nuclear envelope of healthy donor myotubes. Instead, defective PKA nuclear localization was found in EDMD1- myotubes lacking emerin, where AKAP6 was also delocalized. Importantly, recovery of PKA localization to the nuclear rim occurs in genetically corrected EDMD1 myotubes expressing emerin. These results support the hypothesis that emerin, through AKAP6 recruitment to the nuclear envelope, could regulate PKA positioning in the nuclear compartment during muscle differentiation. It is known that PKA targets effectors of cellular mechanosignaling. In particular, PKA stimulates LATS1/2 kinases to phosphorylate the transcription factor YAP on its Ser127 and Ser381 residues, thus avoiding its nuclear translocation [34, 41]. Here, we show YAP nuclear accumulation in EDMD1 muscle cells, where emerin is absent, and PKA nuclear recruitment is lost, whereas rescue of YAP localization was observed in gene edited EDMD1 myoblasts. We hypothesize that defective PKA recruitment to the nuclear rim could contribute to YAP mislocalization to myonuclei due to impaired phosphorylation. Of note, altered signaling through the YAP-mediated Hippo pathway was previously described in muscular laminopathies including EDMD1 [42–45]. Our study further demonstrates that emerin is involved in the correct positioning of SUN proteins and previous studies demonstrate that SUN1 is related to pericentrin localization in myonuclei [31].

The results here described, add to the recently reported evidence that desmin, a muscle-specific intermediate filament and a key cytoskeleton component, is disorganized around the nuclear envelope of laminopathic muscle cells [44]. Thus, we suggest that an altered cytoskeleton remodeling could be a main trigger of EDMD pathogenesis, although the interplay between different cytoskeleton components in laminopathic muscle warrants further investigation. The evidence that mutations of genes encoding different nuclear envelope proteins are responsible for the same pathological phenotype, suggests that the EDMD pathology could be based on a common mechanism involving the LINC complex and altered relocalization of centrosome components to the nuclear envelope in differentiating myoblasts and striated muscle tissue [18, 31].

## Materials and Methods

### Cell culture and skeletal muscle tissue

Human myotube cultures were obtained from muscle biopsies of healthy donors and EDMD1 patients characterized by emerin-null phenotype. The first patient *(*EDMD1 #1), carried an *EMD* mutation in the exon 1 (c.1A>G), a base transition that occurs in the start codon and abolishes the initiation of translation. The muscle biopsy, performed at the age of 5, showed variability in fiber size and shape, with small rounded fibers, fiber splitting and some necrotic fibers with macrophage infiltration. Emerin staining demonstrated absent expression in all myonuclei. Clinically, since the age of 3, the patient presented with diffuse hypotonia, muscle hypotrophy, and joint laxity, along with elevated CK levels. At 7 years of age, the phenotype included mild axial weakness, early scoliosis, pectus excavatum, joint hyperlaxity with mild ankle retraction, and diffuse muscle hypotrophy. Respiratory function was preserved. Cardiac monitoring showed minor rhythm abnormalities, while cardiac ultrasound revealed normal global cardiac function.

The second patient *(*EDMD1 #2), carrying a mutation in *EMD* gene (exon 6) with nucleotide sequence variation in hemizygosis c.650_654dupTGGGC, corresponding to p.Gln219Trpfs*20 mutation. In this patient, the 5 nt insertion in the last exon of *EMD* gene introduces a frameshift and a stop codon after amino acid 218 of the protein. The biopsy, performed at age 11, showed some degenerated muscle fibers and absent emerin expression. Dystrophin, merosin and sarcoglycans were normally expressed. The patient had motor difficulties since age 3, particularly in lifting weights and rising from the floor, with CK levels ranging from 800 to 1400 U/L. At age 5, he showed limb and axial muscle weakness, a tendency to drop the head, mild contractures of the ankles, cervical spine, and elbows, and joint hyperlaxity in the shoulders, knees, and hips. By age 9, a mild restrictive respiratory deficit had developed. At the current age of 15, cardiac evaluation (ECG and cardiac MRI) is normal, with an ejection fraction of 65%. Clinically, he presents with global rigid spine, mild scoliosis, surgically treated severe ankle contractures, and a waddling gait. He is able to climb and descend stairs without support and can stand up with support. For all the studies, we used cells from passage 4 to passage 6 from patients and controls. Controls were age and sex matched. The patient EDMD1#1 is a 5-year-old male, patient EDMD#2 is a male 11 years old, control 1 is a male 11 years old, control 2 is a male 4 years old, control 3 is a male, 9 years old and finally control 4 is a male 13 years old. In addition, we used muscle sections from a female control, aged 8 years.

Biopsies were collected in the BioLaM biobank, approved by the “IOR Ethics Committee” on 05/09/2016. Prot. gen 0018250 - 01-13. All EU and local ethical rules were respected. Cells were grown in Dulbecco’s modified Eagle’s medium (DMEM), supplemented with 20% fetal bovine serum (FBS) (Gibco Life Technology, Thermo Fisher Scientific, Waltham, MA, USA) and antibiotic-anti-mycotic solution (Sigma-Aldrich, St. Louis, MO, USA).

### Antibodies

Antibodies utilized were: anti-emerin, (MONX10804, Monosan, Uden, The Netherlands) used at 1:200 dilution for IF and in situ proximity ligation assay (PLA); anti-pericentrin (ab99341, Abcam, Cambridge, UK; NB 100-6107 Novus Biologicals, Toronto, Canada) used at 1:100 dilution for IF; anti-AKAP6 (#07-087, Upstate, Sigma-Aldrich, St. Louis, MO, USA) used 1:100 for IF and PLA, 1:500 for WB; anti-SUN1 (HPA008346, Sigma-Aldrich, St. Louis, MO, USA); anti-SUN2 (HPA001209, Sigma-Aldrich, St. Louis, MO, USA); anti-PKA RIIa subunit (P-2854, Sigma-Aldrich, St. Louis, MO, USA; P45320, BD Tranduction Laboratories, NJ, USA) used 1:100 for IF and 1:200 for PLA; anti-β-tubulin (Sigma-Aldrich, St. Louis, MO, USA) used 1:200 for IF; anti-perlecan (Millipore, Sigma-Aldrich, St. Louis, MO, USA) used 1:100 for IF; anti-dynein (Ab23905, Abcam, Cambridge, UK) used 1:250 for IF; anti-YAP (sc-101199, Santa Cruz Biotechnology, Dallas, TX, USA) used 1:100 for IF; anti-TOM20 (sc-11415, Santa Cruz Biotechnology, Dallas, TX, USA) used 1:100 for IF; anti-caveolin-3 (BD Tranduction Laboratories, NJ, USA) used 1:200 for IF.

### CRISPR-Cas editing

To correct the mutation of EDMD1 #1, a p.Met1Val variant caused by an A to G transition in the *EMD* exon 1 (c.1A>G), Cytosine Base Editing (CBE) system was used. To remove c.650_654dupTGGGC insertion in the exon 6 of *EMD* gene diagnosed in patient EDMD1 #2 and restore the open reading frame (ORF) of *EMD* gene, we used the SpCas9 nuclease combined with a couple of gRNAs designed in opposite orientation. Strategy and efficiency of CRISPR-mediated correction of the base transition A>G in *EMD* carried by EDMD1#1 cells, and CRISPR-mediated reframing of the *EMD* gene in EDMD1#2 cells, are described in Cattin et al. [46].

### Immunoprecipitation and Western blotting

For immunoprecipitation analysis, differentiated myotubes were lysed in a detergent-IP buffer containing: 50 mM Tris-HCl (pH = 7.5), 150 mM NaCl, 0.1% SDS, 1% NP-40, 1 mM DTT, 1.5 mM MgCl, 20 mM NaF, 1 mM PMSF, and protease and phosphatase inhibitors. For each sample, 500 µg of lysate was incubated at 4 °C overnight with 2,5 µg of anti-emerin (MONX10804, Monosan, Uden, The Netherlands) or non-specific immunoglobulins used as a negative control. After the addition of 30 μl of protein A/G (Santa Cruz Biotechnology, Dallas, TX, USA) for 60 min at 4 °C, the immunoprecipitated proteins were washed 3 times in IP buffer. Later, the samples were added to Laemmli’s buffer, boiled, and subjected to Western blot analysis. Immunoblotted bands were detected by an ECL detection system (Thermo Fisher Scientific, Waltham, MA, USA), and intensity measurement was performed using a Bio-Rad MP Imaging System with Image Lab Touch Software version 3.0.1.14 (Bio-Rad Laboratories, Segrate, Milan, Italy).

### In situ proximity ligation assay

In situ proximity ligation assay (PLA) was performed using Duolink® In Situ Detection Reagents Orange (DUO92007) from Sigma (Sigma-Aldrich, St. Louis, MO, USA) according to manufacturer instructions. The procedure is described in Cenni et al. [44]. The samples were observed by a Nikon Eclipse Ni fluorescence microscope (Nikon, Minato, Tokyo, Japan) equipped with a digital CCD camera using NIS Elements AR 4.3 software. Quantitative analysis of PLA results was performed using Duolink Image Tool software (Sigma-Aldrich, St. Louis, MO, USA) by counting 50 nuclei per sample.

### Immunofluorescence staining

Muscle cells from healthy donors and EDMD1 patients were fixed with methanol at room temperature for 10 minutes or with 4% paraformaldehyde for 15 min (4 °C) plus permeabilization with 0,1% Triton X-100 for 5 min; saturation of non-specific binding sites with 4% of bovine serum albumin (BSA) solution for 20 min a RT was performed. For muscle section, 7 µm-thick non-fixed frozen sections of muscle biopsies from the EDMD1 patients and two age-matched donors were collected on polylisine-coated slides (Thermo Scientific) slides. Coverslips and muscle sections were incubated with primary antibodies overnight at 4 °C O.N or 1 h at RT, and revealed with Cy5, FITC or TRITC-conjugated secondary antibodies diluted 1:200 (incubated for 1 h at RT). Samples were stained with DAPI and mounted with an anti-fading reagent (Molecular Probes Life Technologies).

### Imaging and SIM analysis

Immunofluorescence and PLA analysis, were observed using a Nikon Eclipse Ni epifluorescence microscope with 40x, 60x and 100x objectives (Nikon, Minato, Tokyo, Japan). The images captured with NIS- Elements 4.3 AR software were elaborated using Photoshop CS. SIM analysis of fluorescence and PLA signal was performed using Nikon-Structured Illumination Microscopy (3D N-SIM) (Nikon, Minato, Tokyo, Japan) as described in Santi et al. [47].

### Statistical analysis

For statistical analysis, appropriate tests were chosen according to data distribution. We use a box plot as a graphical method to determine the difference in spread between the groups and to depict the distributions of one or more groups of numeric data. Box plot is represented by plotting individual data points of three independent experiments (*n* = 3), and statistically significant differences between values are indicated with Student’s *t* test (*p < 0.05 or **p < 0.01, *** *p* < 0.001, **** *p* < 0.0001). For measuring a linear correlation between two variables, data were analyzed with Pearson correlation coefficient (*r*), values between 0 and 1 are statistically significant. Microtubule orientation and mitochondrial distribution analysis were performed with Fiji-ImageJ plugin OrientationJ (Biomedical Imaging Group, Lausanne, Switzerland). The coherency value ranges from 0.1 to 1.0, where 1.0 represents the highest coherency and indicates the maximum degree of orientation of the marked structures. The exact sample size (*n*) for each experimental condition is specified in the corresponding figure legends. Data derived from the indicated number of patients (*n* = 2) or non-affected individuals (*n* = 5). Statistical power was calculated using the *sampsizepwr* function in MATLAB R2022a, based on the mean fluorescence intensity of emerin in control and EDMD1 myoblasts. Statistical power ranges from 0 to 1, with 1 indicating maximum power. A value of at least 0.8 is generally considered acceptable. In our study, the statistical power for a two-tailed *t*-test was 0.8738, indicating adequate power.

## Supplementary information

supplemental materials

Original western blot revision

## Data Availability

The raw data supporting the conclusions of this article will be made available by the authors on request.
