# Supplementary material for: Emerin is necessary for microtubule-organizing center translocation to the nuclear envelope of muscle cells
Source: Cell Death Dis. 2026 May 12;17(1):615. doi: 10.1038/s41419-026-08819-6 (PMC13338233; doi:10.1038/s41419-026-08819-6)
Supplement: Supplementary file 1 — supplemental materials [file 41419_2026_8819_MOESM1_ESM.docx]

**Emerin is necessary for microtubule-organizing center translocation to the nuclear envelope of muscle cells**

ELISABETTA MATTIOLI^1,2^*, VITTORIA CENNI^1,2^, PATRIZIA SABATELLI ^1,2^, ELISA SCHENA^1,2^, SPARTACO SANTI^1,2^, CHIARA FIORILLO^3,4^, CLAUDIO BRUNO ^3,5^, ANTONELLA PINI^6^, MELANIA GIANNOTTA^6^, MARCO CAVALLO^7^, COSTANTINO ERRANI^8^,ELEONORA CATTIN^9,10^, DANIELA BENATI ^9,10^ , ALESSANDRA RECCHIA ^9,10^, GIOVANNA LATTANZI^1,2^*.

^1^ CNR Institute of Molecular Genetics “Luigi Luca Cavalli-Sforza”, Unit of Bologna, Bologna, Italy. e.mattioli@area.bo.cnr.it (E.M); [vittoria.cenni@cnr.it](mailto:vittoria.cenni@cnr.it) (V.C.); patrizia.sabatelli@area.bo.cnr.it (P.S.); elisa.schena@cnr.it (E.S.); [spartaco.santi@cnr.it](mailto:spartaco.santi@cnr.it) (S.S.) giovanna.lattanzi@cnr.it (G.L)

^2^ IRCCS Istituto Ortopedico Rizzoli, Bologna, Italy. e.mattioli@area.bo.cnr.it (E.M); [vittoria.cenni@cnr.it](mailto:vittoria.cenni@cnr.it) (V.C.); patrizia.sabatelli@area.bo.cnr.it (P.S.); elisa.schena@cnr.it (E.S.); [spartaco.santi@cnr.it](mailto:spartaco.santi@cnr.it) (S.S.); giovanna.lattanzi@cnr.it (G.L)

^3^ Department of Neurosciences, Rehabilitation, Ophthalmology, Genetics, Maternal and Child Health (DINOGMI), University of Genova, Genova, Italy. chiara.fiorillo@edu.unige.it (C.F.) [claudio.bruno@unige.it](mailto:claudio.bruno@unige.it) (C.B.)

^4^ Child Neuropsychiatry, IRCCS G.Gaslini Institute, Genoa, Italy. chiara.fiorillo@edu.unige.it (C.F.)

^5^ Center of Translational and Experimental Myology, IRCCS Istituto G. Gaslini, 16147 Genova, Italy. [claudio.bruno@unige.it](mailto:claudio.bruno@unige.it) (C.B.)

^6^ Child Neurology and Psychiatry Unit, IRCCS Istituto delle Scienze Neurologiche di Bologna, Bologna, Italy. antonella.pini@isnb.it (A.P.); [melania.giannotta@isnb.it](mailto:melania.giannotta@isnb.it) (M.G.)

^7^ Shoulder-Elbow Surgery Unit, IRCCS Istituto Ortopedico Rizzoli, Bologna, Italy. [marco.cavallo@ior.it](mailto:marco.cavallo@ior.it) (M.C.)

^8^ 3rd Orthopaedic and Traumatologic Clinic prevalently Oncologic. IRCCS Istituto Ortopedico Rizzoli, Bologna, Italy [costantino.errani@ior.it](mailto:costantino.errani@ior.it) (C.E.)

^9^ Department of Life Sciences, University of Modena and Reggio Emilia, Modena, Italy. arecchia@unimore.it (A.R); [eleonora.cattin@unimore.it](mailto:eleonora.cattin@unimore.it) (E.C.); [daniela.benati@unimore.it](mailto:daniela.benati@unimore.it) (D,B.)

^10^ Center for Regenerative Medicine, “Stefano Ferrari” University of Modena and Reggio Emilia, Modena, Italy. arecchia@unimore.it (A.R); [eleonora.cattin@unimore.it](mailto:eleonora.cattin@unimore.it) (E.C.); [daniela.benati@unimore.it](mailto:daniela.benati@unimore.it) (D,B.)

*Corresponding authors, e.mattioli@area.bo.cnr.it ; giovanna.lattanzi@cnr.it.

**Summary**

- **Pericentrin and AKAP6 defective recruitment in EDMD1 myotubes.**
- **Pericentrin and AKAP6 recovered in EDMD1 myotubes after CRISPR editing and emerin rescue.**
- **PKA and AKAP6 colocalization in myonuclei of healthy donor and PKA defective recruitment in the nuclei of EDMD1 myotubes.**
- **Emerin deficiency is associated with an incorrect assembly of the microtubule system and an altered dynein distribution in EDMD1 myotubes.**

**Supplemental figures**

**
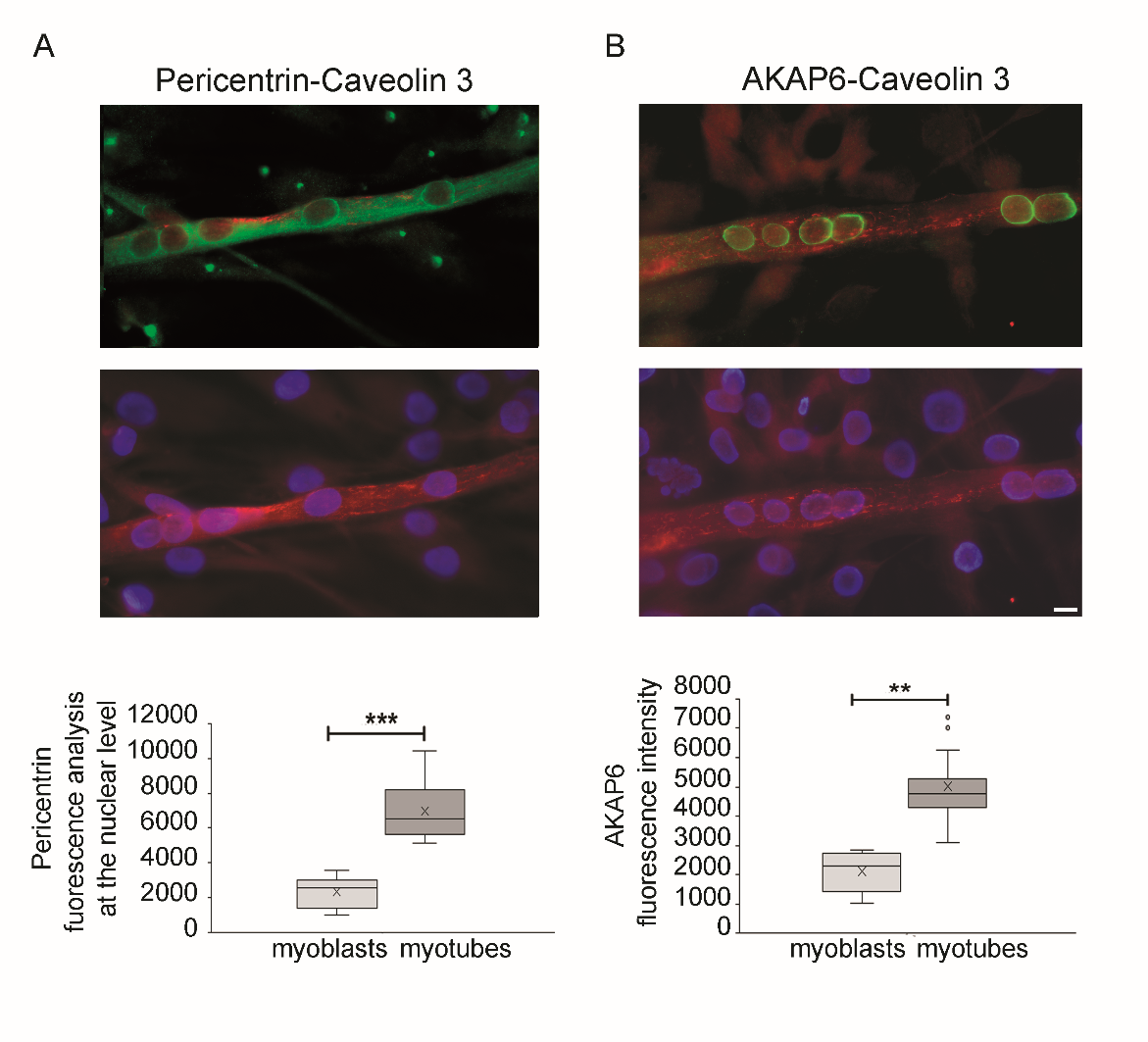
**

**Fig. S1: Pericentrin and AKAP6 recruitment to the nuclear envelope in myotubes.**

**A** Immunofluorescence analysis of pericentrin (green) and caveolin 3 (red) in control myotubes.

Graphical analysis was reported in the box plot graph below. **B** Immunolabeling of AKAP6 (green) and caveolin 3 (red). Mean fluorescence intensity of AKAP6 was reported in the graph below. Caveolin 3 was used as a differentiation marker. DAPI (blue) was used to counterstain cell nuclei. Scale bars, 10 μm. Statistically significant differences between values are indicated with Student’s t-test ( ***p* < 0.01,*** *p* < 0.001). Three biological replicates were used for each experiment (*n*=3) and *n*=20 cells for sample were counted.


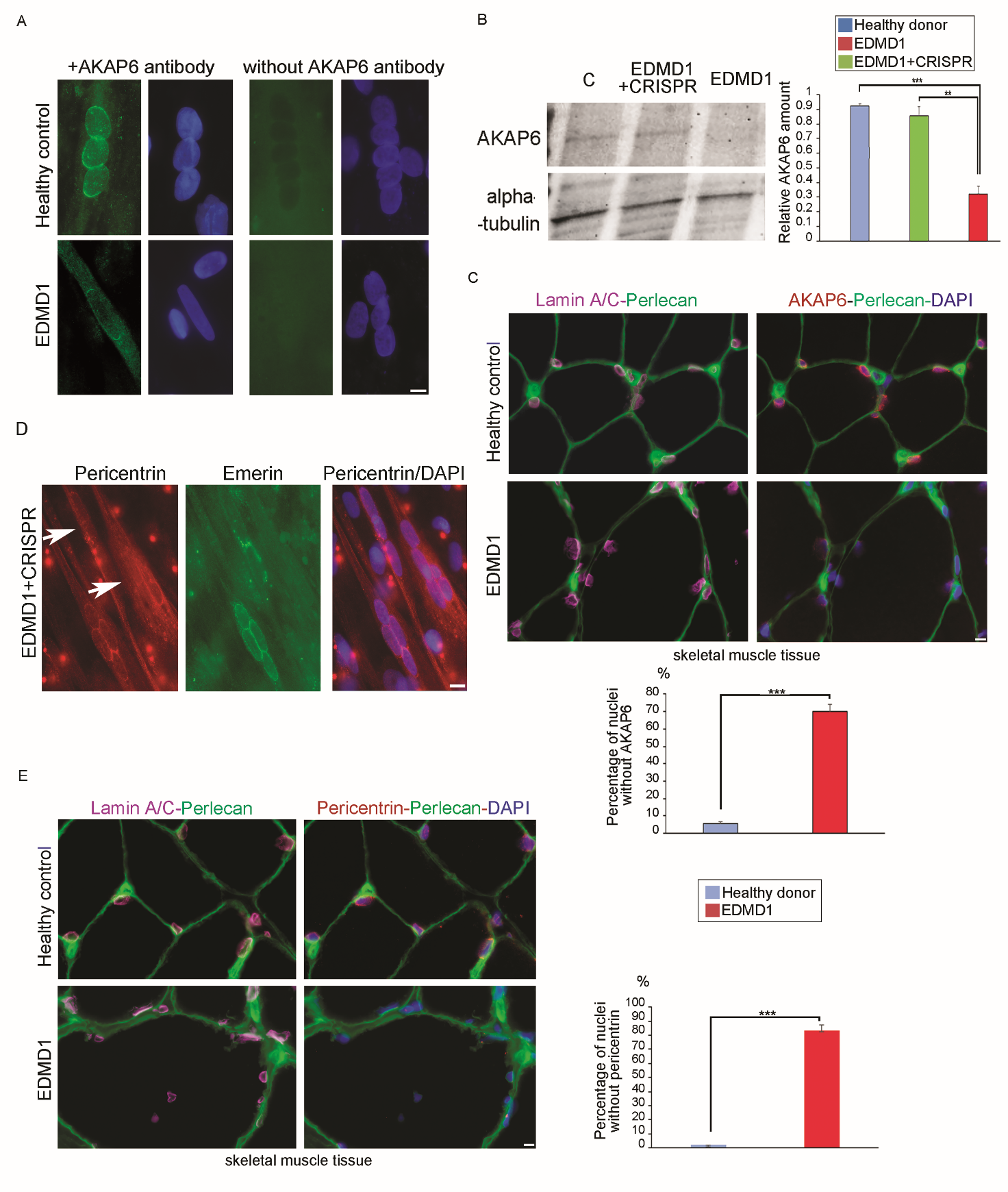


**Fig. S2: AKAP6 reduction in differentiated muscle cells and in skeletal muscle tissue of EDMD1.**

**A** Immunofluorescence analysis of Healthy control and EDMD1 myotubes incubated with primary antibody, anti-AKAP6 (+AKAP6 antibody) and with secondary-only antibody (without AKAP6 antibody). **B** Western blotting analysis of AKAP6 and alpha-tubulin in myotubes lysate of control (c), EDMD1 edited (EDMD+CRISPR) and EDMD1 (EDMD1). Densitometric analysis of AKAP6 normalized on alpha-tubulin is shown on the right. **C** Immunofluorescence analysis of AKAP6 (red) , perlecan (green) and lamin A/C (violet) in skeletal muscle tissue. Percentage of nuclei without AKAP6, inside the muscle fibers, in healthy control and EDMD1 were reported in the graph below. **D** Immunofluorescence analysis of pericentrin (red) and emerin (green) in EDMD1 myotubes subjected to CRISPR editing. White arrows indicate the myotubes do not express emerin, they show reduced pericentrin level. At the other hand in nuclei where emerin expression was restored, pericentrin localization is comparable to heathy donor myotubes. **E** Immunofluorescence analysis of pericentrin (red) , perlecan (green) and lamin A/C (violet) in skeletal muscle tissue. In the graph on the right we shown the percentage of nuclei, inside the muscle fibers, without pericentrin, in control and EDMD1 muscle tissue. Three independent experiments were performed (*n*=3), for muscle tissue samples were counted 40 nuclei (*n*=40) for each experiment and statistically significant differences between values are indicated with Student’s t-test ( ***p* < 0.01 and ****p* < 0.001).

**
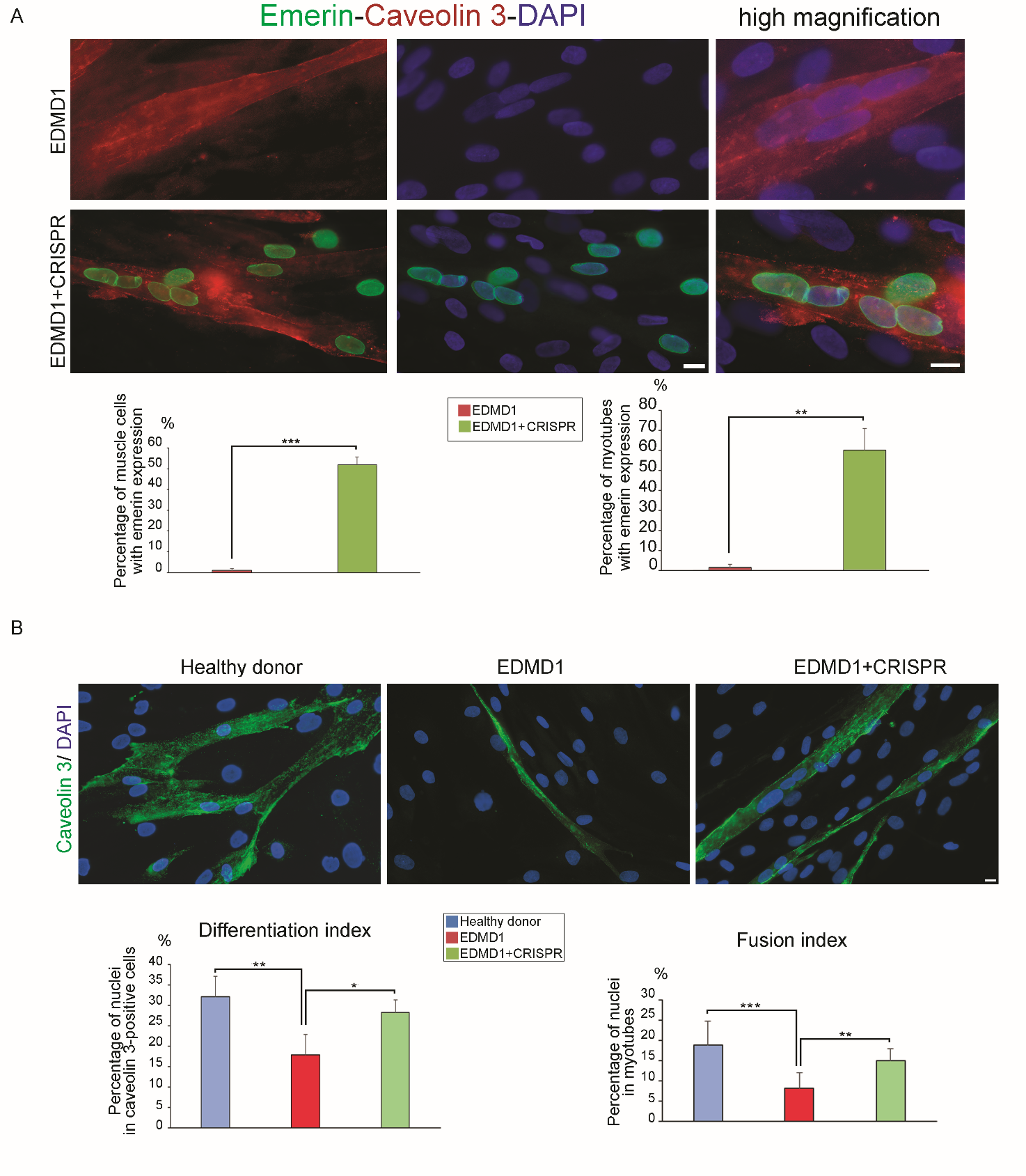
**

**Fig. S3: Emerin restored in EDMD1 muscle cells subjected to CRISPR technology.**

**A** Immunofluorescence analysis of emerin (green) and caveolin 3 (red) in EDMD1 myotubes or EDMD1 myotubes subjected to CRISPR editing. High magnification were reported in the right panels. Percentage of emerin expression were reported in the lower graphs. Nuclei were counterstained with DAPI (blue). Scale bars, 10 μm. **B** Healthy donor, EDMD1 and edited EDMD1 muscle cells were incubated with antibody against caveolin 3 (green) and stained for DAPI (blu). Differentiation index (%) is calculated by dividing the number of nuclei in multinucleated Caveolin 3-positive myotubes, plus the number of nuclei of mononucleate caveolin 3-positive cells by the total number of nuclei present in a field of view. The total multiplied by 100. The fusion index (%) is calculated by dividing the number of multinucleated caveolin 3-positive myotubes (> 2 nuclei) by the total number of nuclei present in a field of view. The total multiplied by 100. Three

independent experiments were performed (*n*=3) and statistically significant differences between values are indicated with Student’s t-test ( ***p* < 0.01 and ****p* < 0.001).


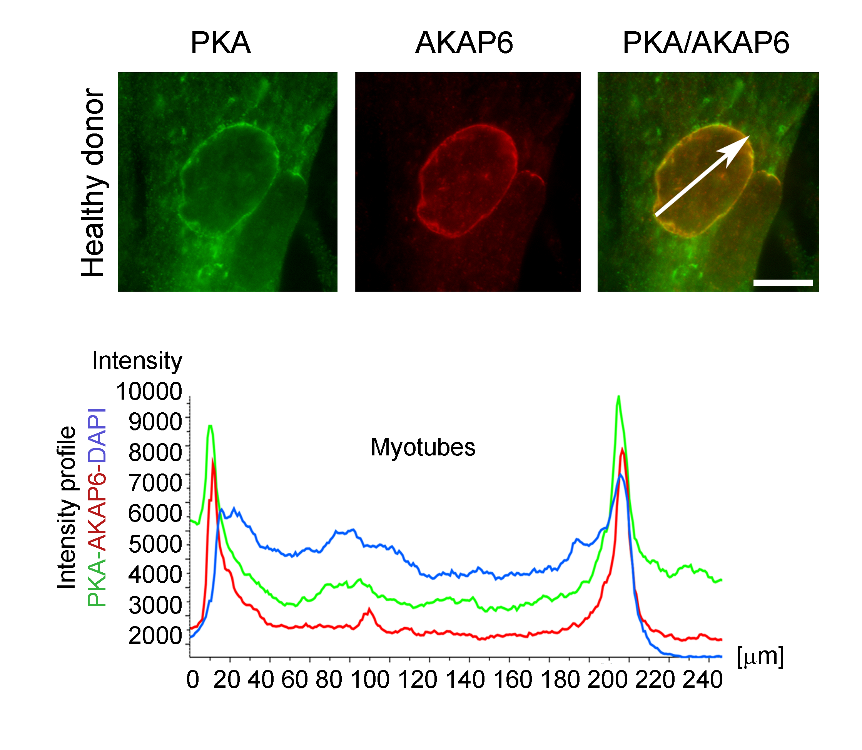


**Fig. S4: PKA and AKAP6 colocalization in myonuclei of healthy donor.**

Immunofluorescence analysis of PKA (green) and AKAP6 (red) and merge image in the panel on the right. In the lower graph fluorescence intensity profile of PKA (green), AKAP6 (red) and DAPI (blu) in a representative nucleus of control myotube (white arrow).
