# Supplementary figures and images for: Emerin is necessary for microtubule-organizing center translocation to the nuclear envelope of muscle cells
Source: Cell Death Dis. 2026 May 12;17(1):615. doi: 10.1038/s41419-026-08819-6 (PMC13338233; doi:10.1038/s41419-026-08819-6)

**Raw data**

Fig. 3C Co-Immunoprecipitation experiment


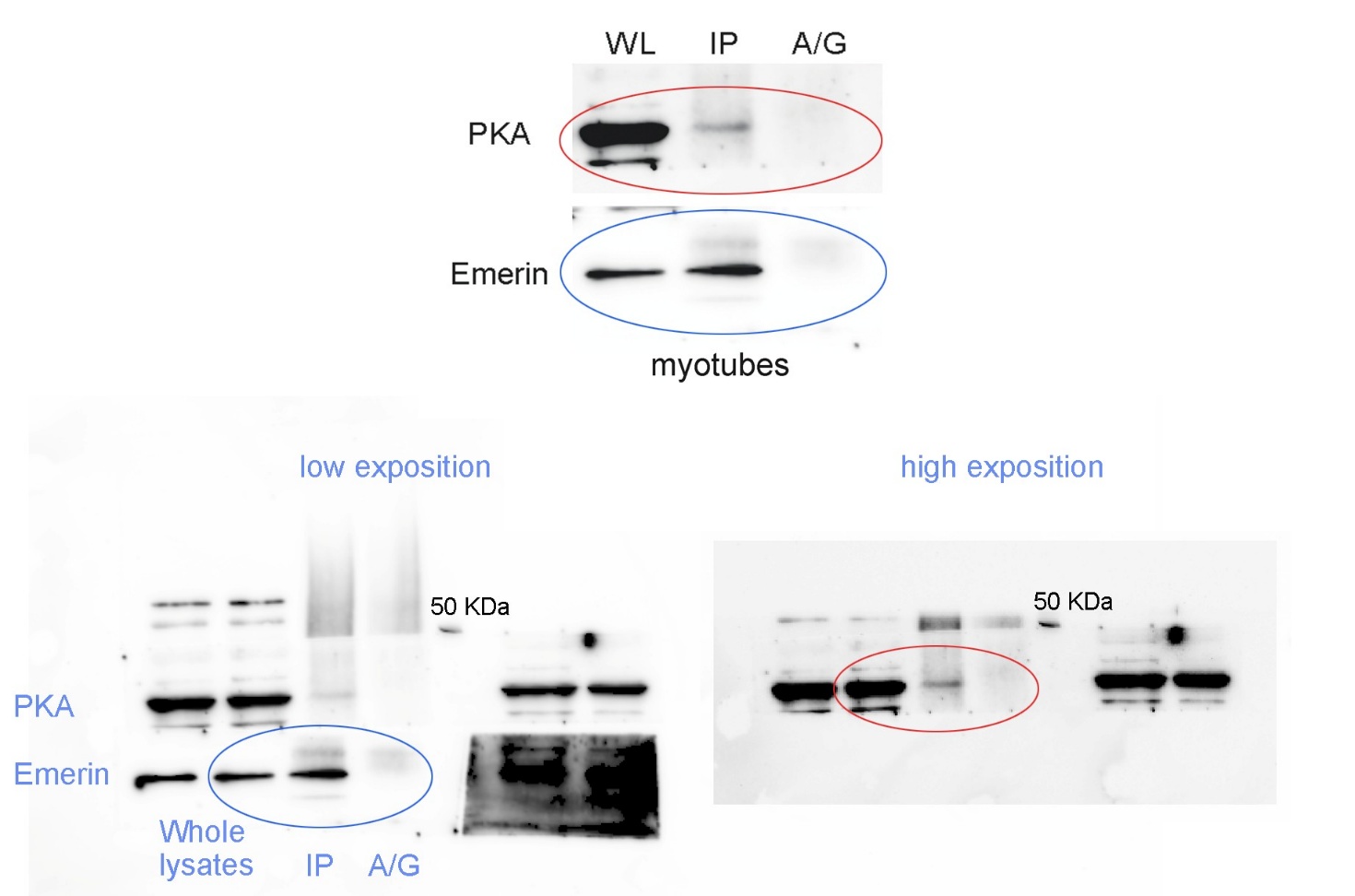


Fig. S2B Western blotting AKAP6


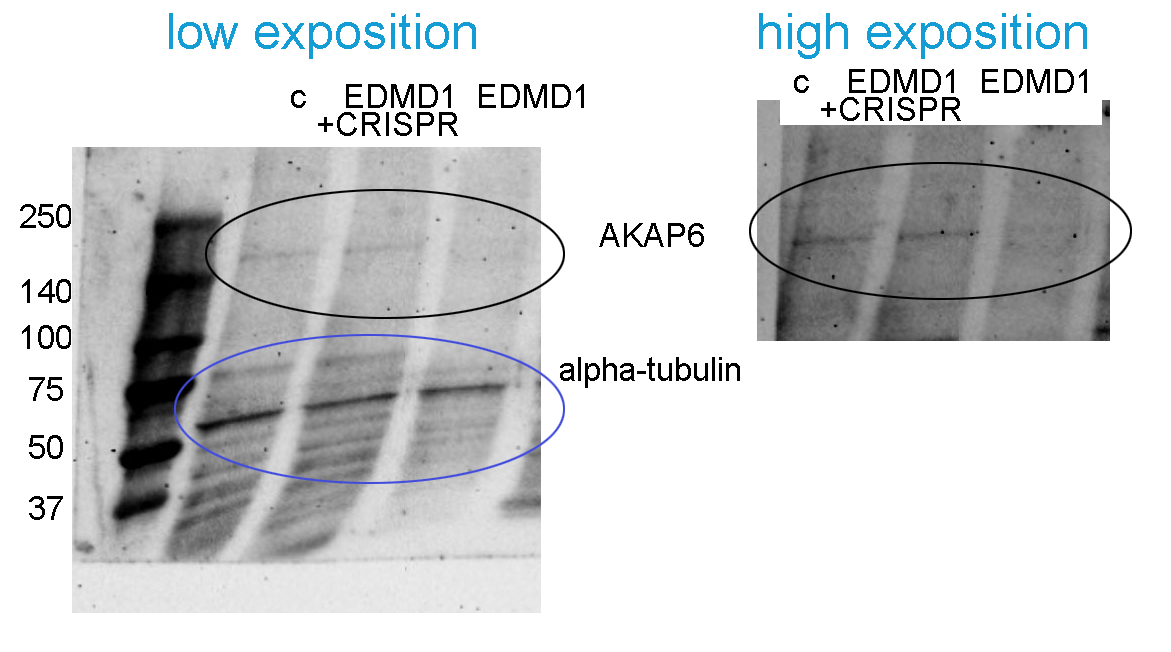

Supplement: Supplementary file 2 — Original western blot revision [file 41419_2026_8819_MOESM2_ESM.docx]
